# Supplementary material for: The microprotein C16orf74/MICT1 promotes thermogenesis in brown adipose tissue
Source: EMBO J. 2025 May 12;44(12):3381–412. doi: 10.1038/s44318-025-00444-x (PMC12170882; doi:10.1038/s44318-025-00444-x)
Supplement: Supplementary file 3 — Expanded View Figures [file 44318_2025_444_MOESM3_ESM.pdf]

# Expanded View Figures

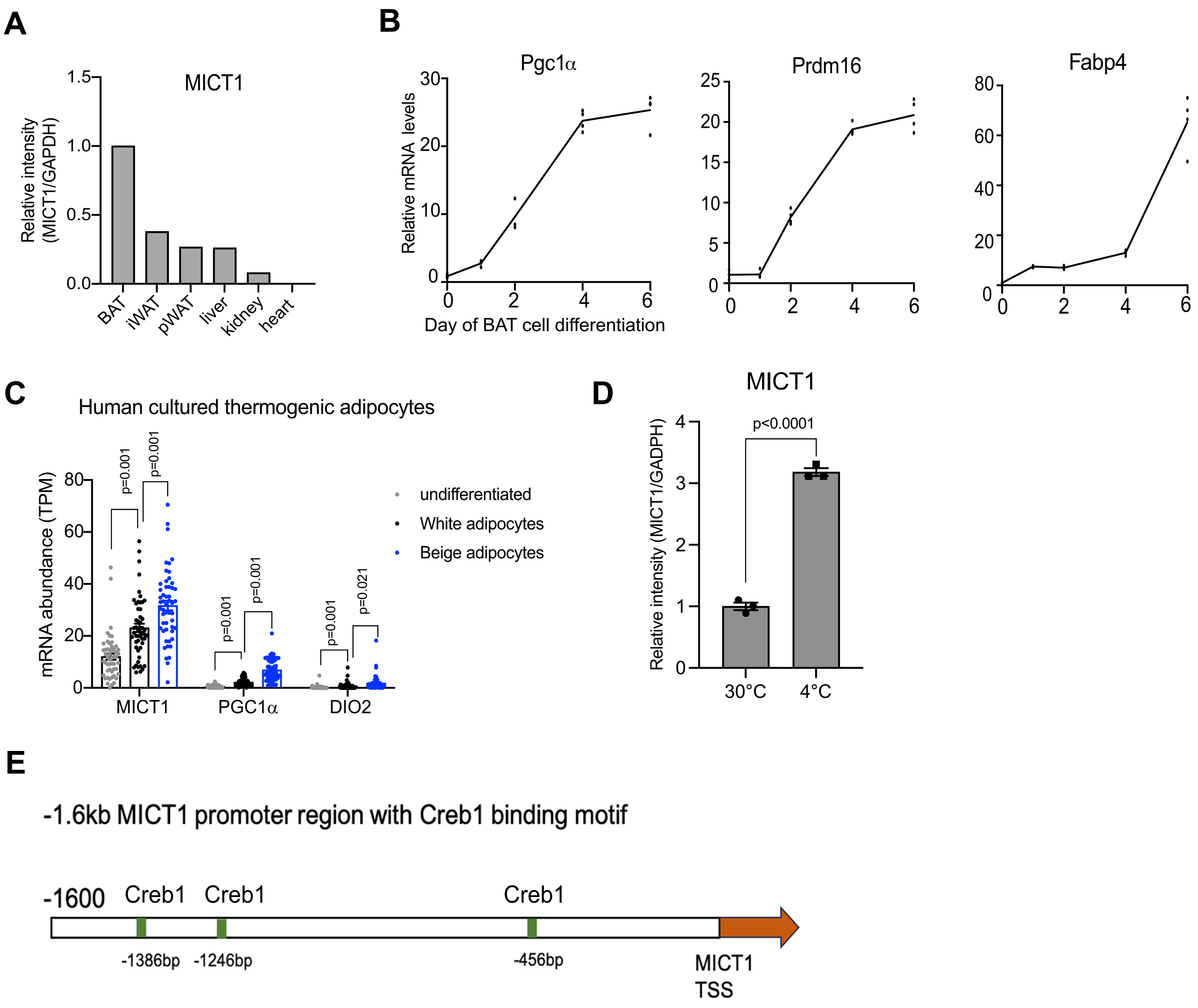


**Figure EV1. MICT1 is a microprotein highly enriched in brown adipocytes.**

(**A**) MICT1 protein quantification for various mouse tissues. (**B**) RT-qPCR for during the course of BAT cell differentiation. (**C**) Expression of MICT1 in human cultured thermogenic adipocytes, from publicly available RNA-seq data ($n = 50$, White adipocytes MICT1: $P = 0.001$, Beige adipocytes MICT1: $P = 0.001$, White adipocytes PGC1α: $P = 0.001$, Beige adipocytes PGC1α: $P = 0.001$, White adipocytes DIO2: $P = 0.001$). (**D**) MICT1 protein quantification for mice housed at either 30 °C or 4 °C ($n = 3$) (**E**) Schematic of CREB sites in the MICT1 promoter. Data is expressed as means ± standard errors of the means (SEM) of indicated number of biological replicates. The statistical differences in mean values were assessed by Student's *t* test.

© The Author(s)

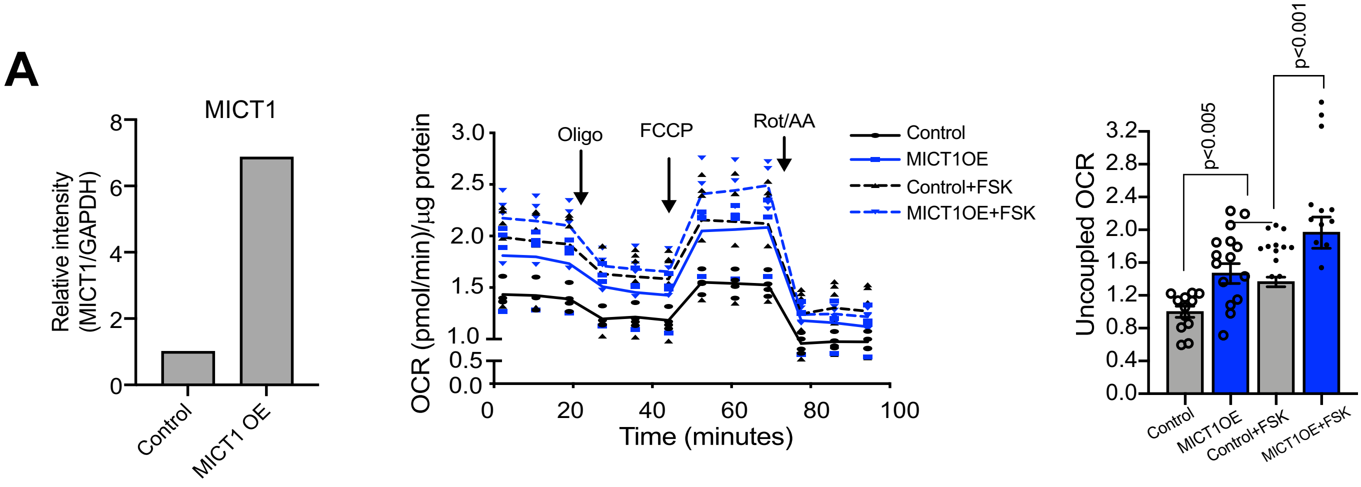


**Figure EV2. MICT1 impact on thermogenesis in cultured brown adipocytes.**

(**A**) (Left) MICT1 protein quantification in MICT1 OE BAT cells. (Right) OCR measured in MICT1 OE BAT cells that were treated with FSK, and relative uncoupled OCR under oligomycin (0.5 µM) ($n = 12$, MICT1 OE: $P = 0.0041$, MICT1 + FSK: $P = 0.0009$). Data is expressed as means ± standard errors of the means (SEM) of indicated number of biological replicates. The statistical differences in mean values were assessed by Student's *t* test.

 © The Author(s)

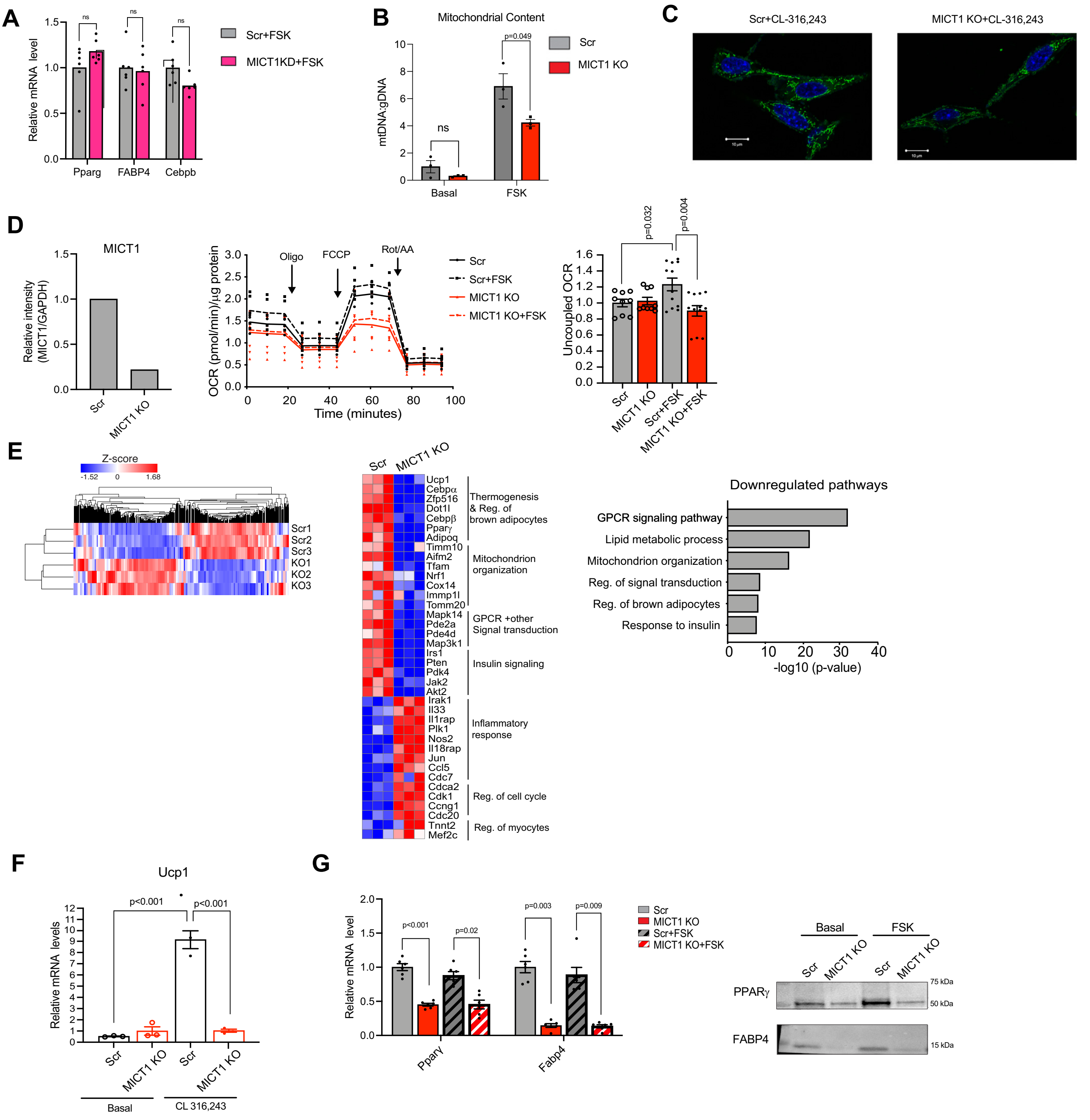


**Figure EV3. MICT1 ablation suppresses thermogenesis in cultured brown adipocytes.**

(**A**) RT-qPCR in MICT1 KD cells ($n = 6$). (**B**) Ratio of mtDNA:gDNA of Scr and MICT1 KO cells in the basal and FSK stimulated condition ($n = 3$, MICT1 KO + FSK: $P = 0.0495$). (**C**) (Left) MICT1 protein quantification in MICT1 KO pool cells. (Right) OCR measured in MICT1 KO pools that were treated with FSK, and relative uncoupled OCR under oligomycin (0.5 μM). (**D**) (Left) Hierarchical clustering of RNA-seq using differentiated MICT1-KO pools. (Middle) Heatmap showing changes in gene expression in the Scr and MICT1-KO pools. (Right) Representative top GO terms of downregulated genes identified by differential expression analysis. (**E**) (Left) RT-qPCR for indicated genes in MICT1 KO pools in the basal condition. (Right) RT-qPCR for Ucp1 in MICT1 KO-pools in the basal and CL-316,243 treated conditions. (**F**) RT-qPCR for MICT1 in Scr and MICT1 KO cells in the basal and CL-316,243-stimulated conditions ($n = 3$, Scr+FSK: $P < 0.0001$, MICT1 KO + FSK: $P < 0.0001$). (**G**) RT-qPCR ($n = 6$, MICT1 KO Ppary: $P < 0.0001$) and IB for Ppary and Fabp4 in Scr and MICT1 KO cells in the basal and FSK-stimulated conditions. Data is expressed as means ± standard errors of the means (SEM) of indicated number of biological replicates. The statistical differences in mean values were assessed by Student's *t* test.

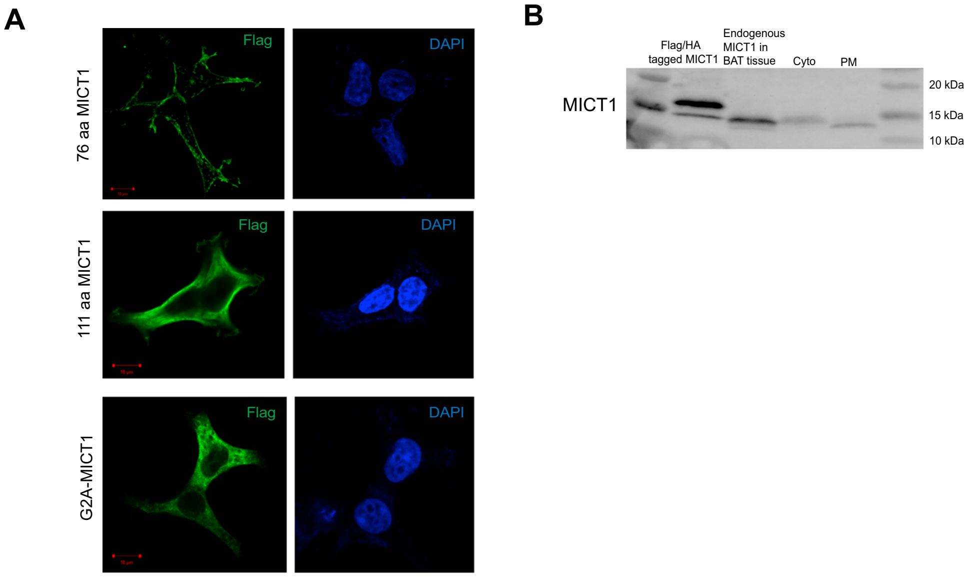


**Figure EV4. Plasma membrane localization of MICT1 and its interaction with PP2B.**

(**A**) IF images of HEK293FT cells overexpressing 76 aa MICT1 (scale bar: 10 μm), 111 aa MICT1 (scale bar: 10 μm), or G2A-MICT1 (scale bar: 10 μm). (**B**) IB for MICT1 in lysates from HEK293FT cells overexpressing Flag/HA tagged MICT1 (first lane) and endogenous MICT1 in mouse BAT (second lane). MICT1 in the cytosol and plasma membrane of mouse BAT (third and fourth lane).

 © The Author(s)

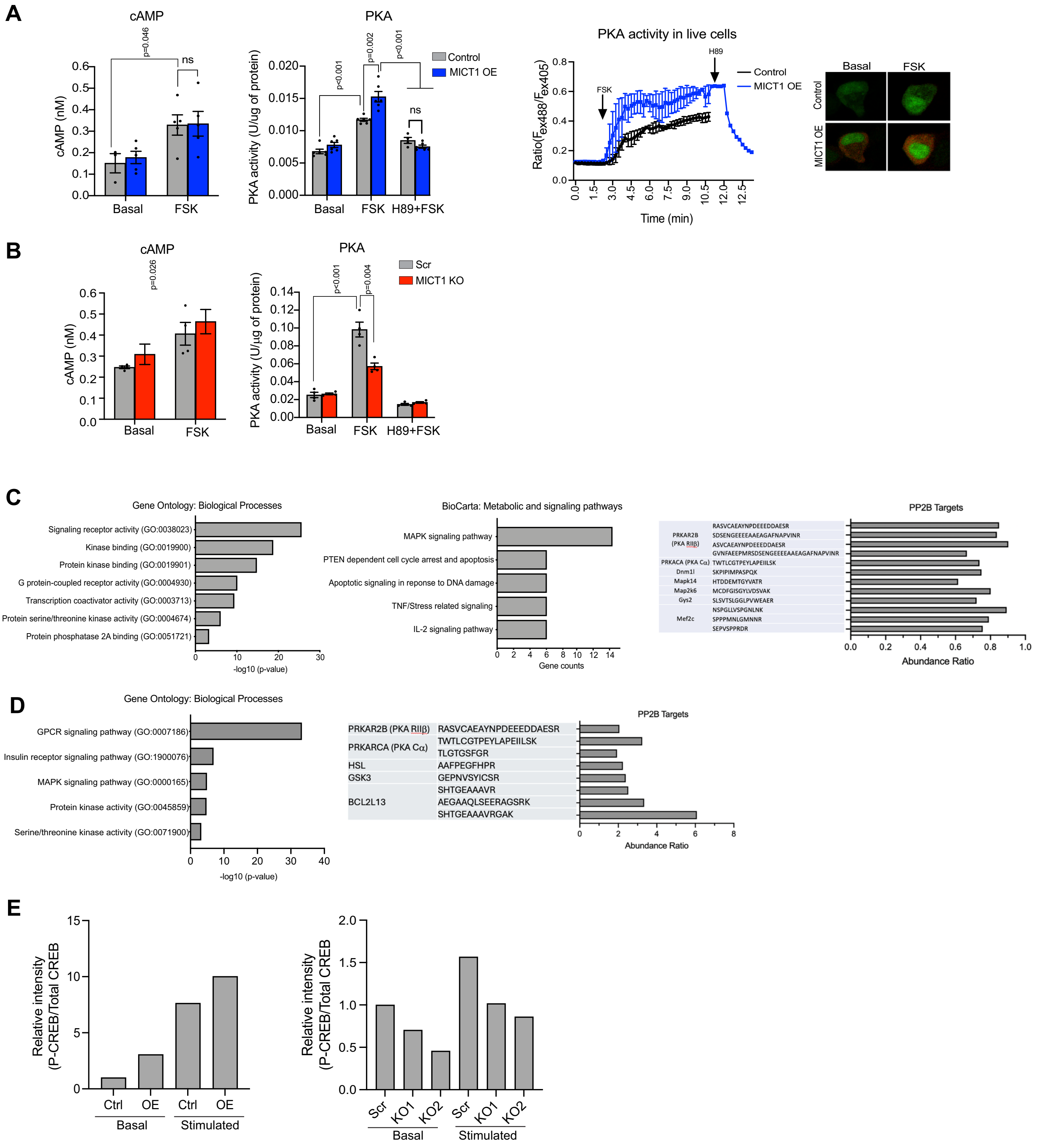
A
cAMP
cAMP (nM)
p=0.046
ns
Basal
FSK
PKA
PKA activity (U/ug of protein)
p<0.001
p=0.002
p<0.001
ns
Control
MICT1 OE
Basal
FSK
H89+FSK
PKA activity in live cells
Ratio(Fex488/Fex405)
FSK
H89
Control
MICT1 OE
Time (min)
Basal
FSK
Control
MICT1 OE
B
cAMP
cAMP (nM)
p=0.026
Basal
FSK
PKA
PKA activity (U/µg of protein)
p<0.001
p=0.004
Scr
MICT1 KO
Basal
FSK
H89+FSK
C
Gene Ontology: Biological Processes
Signaling receptor activity (GO:0038023)
Kinase binding (GO:0019900)
Protein kinase binding (GO:0019901)
G protein-coupled receptor activity (GO:0004930)
Transcription coactivator activity (GO:0003713)
Protein serine/threonine kinase activity (GO:0004674)
Protein phosphatase 2A binding (GO:0051721)
-log10 (p-value)
BioCarta: Metabolic and signaling pathways
MAPK signaling pathway
PTEN dependent cell cycle arrest and apoptosis
Apoptotic signaling in reponse to DNA damage
TNF/Stress related signaling
IL-2 signaling pathway
Gene counts
PP2B Targets
PRKAR2B (PKA RIIβ)
RASVCAEAYNPDEEEDDAESR
SDSENGEEEEAAEAGAFNAPVINR
ASVCAEAYNPDEEEDDAESR
GVNFAEEPMRSDSENGEEEEAAEAGAFNAPVINR
PRKACA (PKA Cα)
TWTLCGTPEYLAPEIILSK
Dnm1l
SKPIPIMPASPQK
Mapk14
HTDDEMTGYVATR
Map2k6
MCDFGISGYLVDSVAK
Gys2
SLSVTSLGGLPVWEAER
Mef2c
NSPGLLVSPGNLNK
SPPPMNLGMNNR
SEPVSPPRDR
Abundance Ratio
D
Gene Ontology: Biological Processes
GPCR signaling pathway (GO:0007186)
Insulin receptor signaling pathway (GO:1900076)
MAPK signaling pathway (GO:0000165)
Protein kinase activity (GO:0045859)
Serine/threonine kinase activity (GO:0071900)
-log10 (p-value)
PP2B Targets
PRKAR2B (PKA RIIβ)
RASVCAEAYNPDEEEDDAESR
PRKARCA (PKA Cα)
TWTLCGTPEYLAPEIILSK
TLGTGSFGR
HSL
AAFPEGFHPR
GSK3
GEPNVSYICSR
BCL2L13
SHTGEAAAVR
AEGAAQLSEERAGSRK
SHTGEAAAVRGAK
Abundance Ratio
E
Relative intensity (P-CREB/Total CREB
Ctrl
OE
Ctrl
OE
Basal
Stimulated
Relative intensity (P-CREB/Total CREB
Scr
KO1
KO2
Scr
KO1
KO2
Basal
Stimulated

**Figure EV5. MICT1 potentiates PKA activity.**

(**A**) (Left) Control and MICT1OE in differentiated BAT cells. cAMP levels and PKA activity were measured in the basal condition or after FSK treatment. Cell permeable H89 was used to verify PKA activity ($n = 4$, Control+FSK: $P = 0.0001$, MICT1 OE + H89 + FSK: $P = 0.0001$). (Right) AKAR images of MICT1 OE cells that were treated with FSK. (**B**) Differentiated Scr or MICT1 KO-pools were used to measure cAMP levels and PKA activity in the basal condition or after FSK treatment ($n = 4$, Scr+FSK: $P < 0.0001$). (**C**) Phosphoproteomics analysis of MICT1-CRISPR KO brown adipocytes that were treated with FSK. (Left) Gene Ontology and pathway analysis by BioCarta indicate that PKA pathway is the top signaling pathway affected by MICT1 ablation. (Right) Known PP2B targets with significantly decreased phosphorylation abundance ratio and annotated sequences. (**D**) Phosphoproteomics analysis of MICT1 OE brown adipocytes that were treated with CL-316,243. (Left) Gene Ontology GPCR signaling pathway is the top signaling pathway affected by MICT1 overexpression. (Right) Known PP2B targets with significantly increased phosphorylation abundance ratio and annotated sequences. (**E**) P-CREB protein quantification for MICT1 OE (left) and MICT1-KO pools (right). Data is expressed as means ± standard errors of the means (SEM) of indicated number of biological replicates. The statistical differences in mean values were assessed by Student's *t* test.

 © The Author(s)

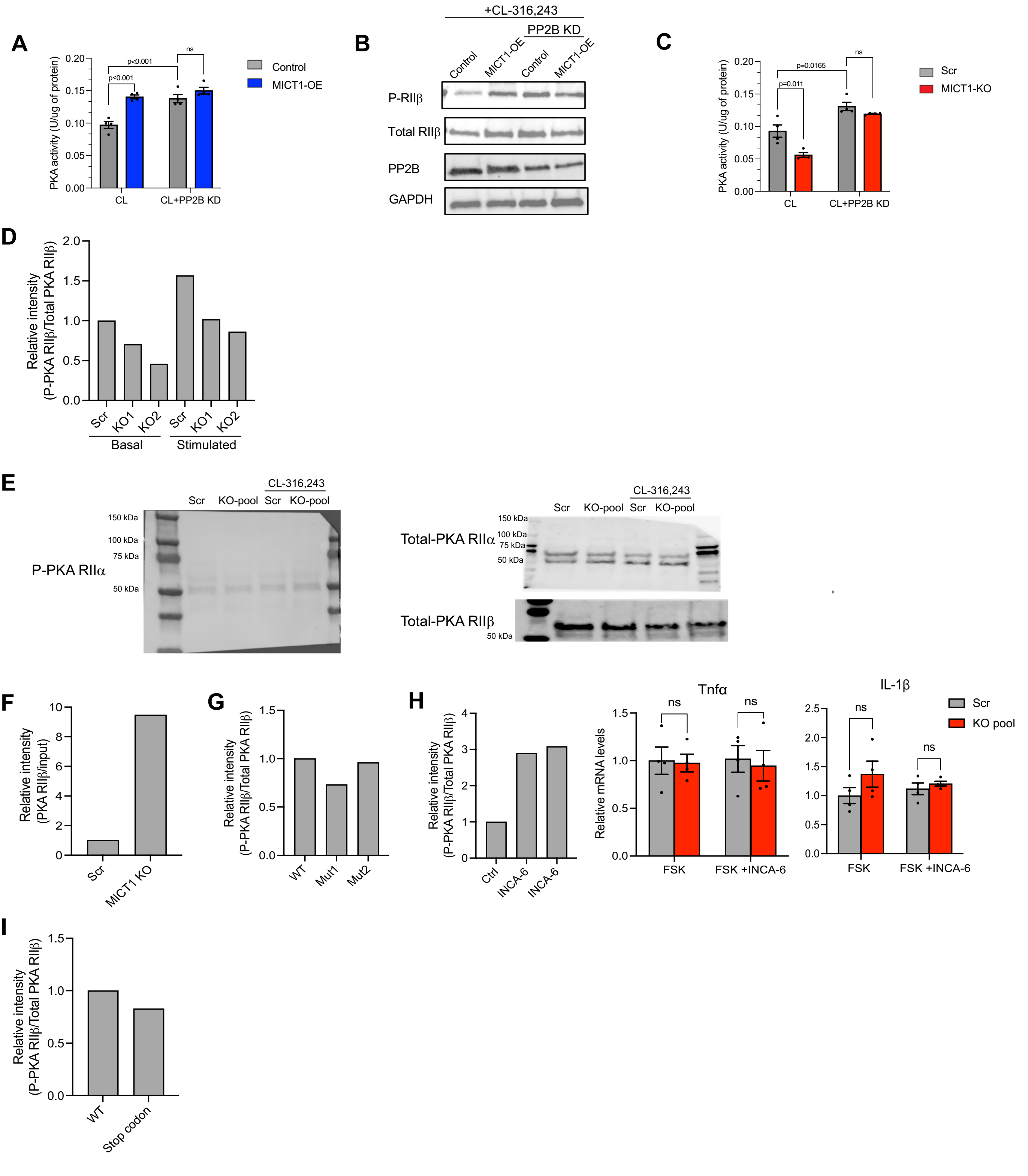
A
PKA activity (U/ug of protein)
p<0.001
p<0.001
ns
Control
MICT1-OE
CL
CL+PP2B KD
B
+CL-316,243
PP2B KD
Control
MICT1-OE
Control
MICT1-OE
P-RIIβ
Total RIIβ
PP2B
GAPDH
C
PKA activity (U/ug of protein)
p=0.0165
p=0.011
ns
Scr
MICT1-KO
CL
CL+PP2B KD
D
Relative intensity (P-PKA RIIβ/Total PKA RIIβ)
Scr KO1 KO2 Scr KO1 KO2
Basal
Stimulated
E
CL-316,243
Scr KO-pool Scr KO-pool
P-PKA RIIα
150 kDa
100 kDa
75 kDa
50 kDa
Total-PKA RIIα
Total-PKA RIIβ
F
Relative intensity (PKA RIIβ/input)
Scr
MICT1 KO
G
Relative intensity (P-PKA RIIβ/Total PKA RIIβ)
WT
Mut1
Mut2
H
Relative intensity (P-PKA RIIβ/Total PKA RIIβ)
Ctrl
INCA-6
INCA-6
Tnfα
Relative mRNA levels
ns
FSK
FSK +INCA-6
IL-1β
Scr
KO pool
I
Relative intensity (P-PKA RIIβ/Total PKA RIIβ)
WT
Stop codon

**Figure EV6. Plasma membrane MICT1-PP2B interaction controls RIIβ dephosphorylation for potentiation of PKA activity and thermogenesis.**

(**A**) PKA activity ($n = 4$, MICT1 OE + CL: $P = 0.0004$, Control+CL + PP2B KD: $P < 0.0004$) and (**B**) IB of CL-316,243 stimulated control and MICT1 OE brown adipocytes with or without PP2B. (**C**) PKA activity of CL-316,243 stimulated Scr and MICT1 KO brown adipocytes with or without PP2B KD ($n = 4$). (**D**) P-PKA RIIβ protein quantification for MICT1 OE (left) and MICT1-KO pools (right). (**E**) IB of MICT1-CRISPR KO brown adipocytes that were treated with CL-316,243. (**F**) Total-PKA RIIβ protein quantification for Scr and MICT1-KO pool lysates that were pulled down with PP2B antibody. (**G**) P-PKA RIIβ protein quantification for MICT1 mutants. (**H**) (Left) P-PKA RIIβ protein quantification for INCA-6 injected BAT lysates. (Right) RT-qPCR for IL-1β and Tnfα in FSK-stimulated MICT1-KO BAT cells that were treated with vehicle or INCA-6 (5μM) for 1 h ($n = 4$). (**I**) P-PKA RIIβ protein quantification for MICT1 with stop codon mutation. Data is expressed as means ± standard errors of the means (SEM) of indicated number of biological replicates. The statistical differences in mean values were assessed by Student's *t* test.

 © The Author(s)

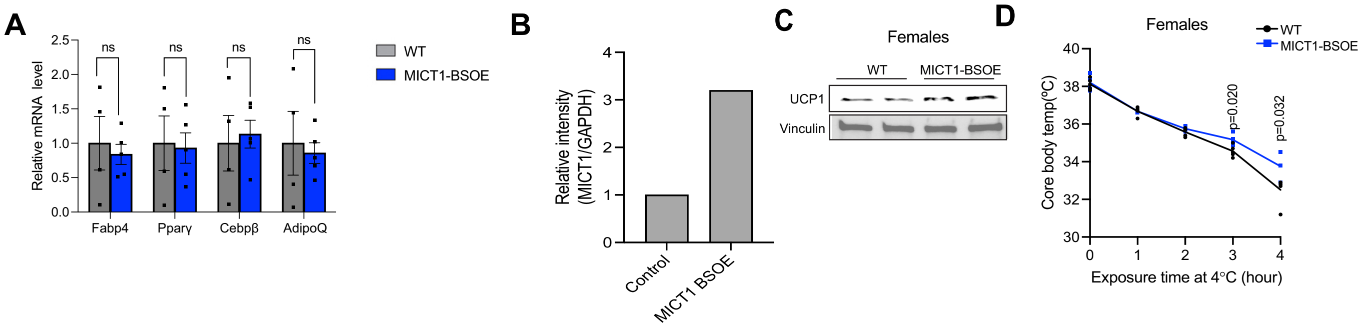


**Figure EV7. MICT1 overexpression in BAT in mice promotes thermogenesis, preventing obesity and insulin resistance.**

(**A**) RT-qPCR for adipogenic genes in BAT of WT and MICT1-BSOE mice ($n = 4$). (**B**) MICT1 protein quantification for MICT1-BSOE mice. (**C**) IB for MICT1 and UCP1 in BAT from MICT1-BSOE and control female mice. (**D**) Core body temperature measured in 13-wk-old female mice at 4 °C at indicated time points ($n = 4$). Data is expressed as means ± standard errors of the means (SEM) of indicated number of biological replicates. The statistical differences in mean values were assessed by Student's *t* test.

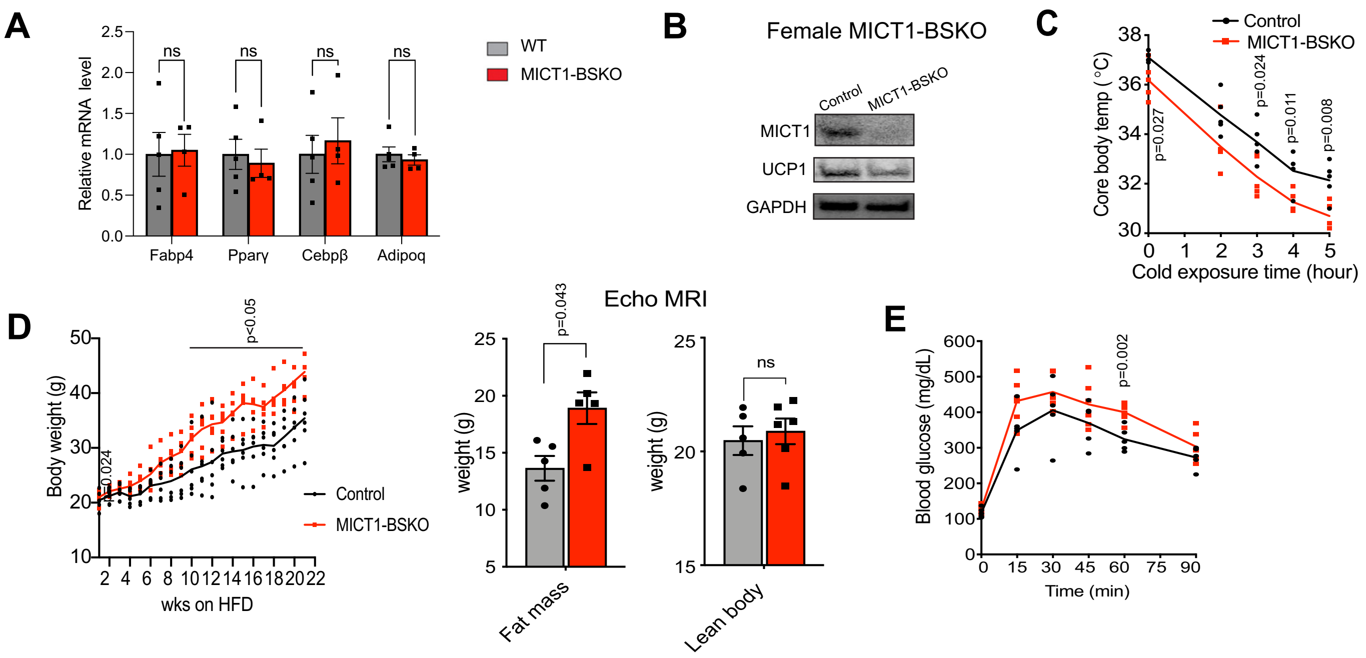


**Figure EV8. MICT1 ablation in BAT in mice reduces thermogenic capacity to gain adiposity.**

(**A**) RT-qPCR for adipogenic genes in BAT of WT and MICT1-BSKO mice ($n = 4$). (**B**) IB for MICT1 and UCP1 in BAT from MICT1-BSKO and control female mice. (**C**) Core body temperature measured in 13-wk-old female mice at 4 °C at indicated time points ($n = 4$ mice per group). (**D**) Body weights and body composition assessed by EchoMRI of control and MICT1-BSKO female mice on HFD ($n = 6$). (**E**) GTT of MICT1-BSKO female mice ($n = 5$). Data is expressed as means ± standard errors of the means (SEM) of indicated number of biological replicates. The statistical differences in mean values were assessed by Student's *t* test.

 © The Author(s)
